# Going for Gaps in Glaucoma

**DOI:** 10.3390/jcm12175494

**Published:** 2023-08-24

**Authors:** Barbara Cvenkel, Miriam Kolko

**Affiliations:** 1 Department of Ophthalmology, University Medical Centre Ljubljana, 1000 Ljubljana, Slovenia; barbara.cvenkel@gmail.com; 2 Faculty of Medicine, University of Ljubljana, 1000 Ljubljana, Slovenia; 3 Department of Drug Design and Pharmacology, University of Copenhagen, 2100 Copenhagen, Denmark; 4 Department of Ophthalmology, Copenhagen University Hospital, Rigshospitalet, 2600 Glostrup, Denmark

Glaucoma is the second leading cause of blindness in people over 50 years of age worldwide, and with the ageing population, this number will continue to rise, resulting in a reduced quality of life for these people and an increased social and economic burden on society [1,2]. Since visual impairment due to glaucoma is preventable, timely detection and treatment is critical. This Special Issue of the *Journal of Clinical Medicine* (JCM) contains 10 articles, of which 6 are research articles and 4 are review articles, addressing issues of diagnosis, treatment and new perspectives in the field of glaucoma. 

In a paper on rapid campimetry, a novel screening method for glaucoma diagnosis, it was shown to be comparable and even better than 10-2 standard automated perimetry in detecting central macular scotomas [3]. It uses a bright, fast-moving target on a high-contrast background that is perceived as interrupted in the scotoma area. From a distance of 40 cm, the diameter of the moving target depends on the distance to the fixation point and increases with increasing distance from the fixation point. The method is promising for glaucoma screening in the future. It is fast and can be used on a commercially available computer connected to the internet. 

Telehealth, i.e., the provision of health care to patients remotely using voice and image communication via a computer or smartphone, was widely used during the coronavirus pandemic. To ensure adequate care for the growing population of glaucoma patients, telehealth and new devices that enable the detection and monitoring of glaucoma will become increasingly important in the future. This review provides an overview of available devices for intraocular pressure measurement, perimetry and fundus photography in a home setting, as well as implemented telehealth programmes for glaucoma screening, monitoring and assessment of glaucoma severity for treatment planning [4]. Ophthalmologists can review results remotely and stratify patients, particularly those with mild to moderate glaucoma and suspected glaucoma, while those with uncontrolled or severe glaucoma should receive an in-person visit. The telehealth approach is cost-effective and particularly beneficial for patients with limited access to healthcare, i.e., in rural areas.

Many eye departments in hospitals are struggling with capacity problems because the increase in newly diagnosed cases is not matched by a proportional increase in the number of ophthalmologists. This inevitably leads to limited access for new patients and an increase in the time between follow-up appointments, leading to the risk that the progression of glaucoma is not detected in time. One solution for the existing number of ophthalmologists is to increase efficiency by offering alternative methods for a safe glaucoma care setting. Simons and colleagues have investigated two alternative schemes for glaucoma care within a hospital, namely shared care and virtual clinics [5]. Both shared care and virtual clinics were found to be safe, cost-effective and acceptable for the care of stable glaucoma patients at low risk of vision loss [5]. The most common non-medical staff involved were optometrists. Patients appreciated the shorter waiting times and did not feel disadvantaged by not seeing a doctor. These two alternative approaches are promising and allow ophthalmologists to assess high-risk patients more quickly without increasing the time intervals for other low-risk glaucoma patients.

Macular pigment plays an important role in visual function and protects the retina from oxidative damage. Macular pigment and glaucoma may be linked through microvascular and oxidative stress processes, both of which have been implicated in the pathogenesis of glaucoma. The Montrachet population-based study, which included participants aged 75 years and older, compared macular pigment optical density and its distribution between eyes with primary open-angle glaucoma and control eyes without optic neuropathy [6]. The macular pigment optical density was determined via the two-wave autofluorescence method using the Heidelberg Retina Angiograph (HRA; Heidelberg Engineering, Heidelberg, Germany) and the macular pigment spatial distribution was generated from the HRA graphs using modified software. Of the 601 eyes, 48 eyes had primary open-angle glaucoma. There were no differences in the macular pigment optical density and its spatial distribution between eyes with primary open-angle and control eyes.

In recent years, the availability and use of psychopharmacological treatments have increased. Ciobanu and co-workers summarise the current knowledge on the risk of psychotropic drug-induced increases in intraocular pressure [7]. Clinicians should be aware of the possibility of psychotropic drug-induced glaucoma and monitor at-risk patients closely, especially if treatment includes tricyclic antidepressants, benzodiazepines and topiramate.

Adherence to IOP-lowering treatment is critical in chronic diseases such as glaucoma, and poor adherence has been associated with faster disease progression [8]. A review paper discusses several strategies to improve adherence, many of which are still in clinical trials [9]. These strategies aim to reduce or avoid the need for eye drops and their side effects. Monitoring devices and smart drug delivery systems, sustained drug delivery systems, lasers, and minimally invasive glaucoma procedures with and without a device in combination with cataract surgery are used for this purpose.

Using an in vitro model, Freiberg and co-workers showed that preservative-free latanoprost ophthalmic solutions differed significantly in their physicochemical properties, including pH, osmolality and surface tension [10]. However, this had no effect on goblet cell viability or mucin release. Future clinical studies are needed to evaluate the long-term efficacy and safety of preservative-free eye drops with different physicochemical properties.

Selective laser trabeculoplasty (SLT) is an established method for lowering intraocular pressure as a first-line treatment or as an adjunct treatment. Treatment with topical steroids or non-steroidal anti-inflammatory drops after SLT is controversial. A retrospective review compared the reduction in intraocular pressure between patients who received topical steroid eye drops for a short time after SLT and those who did not [11]. The success rate was similar in both groups, showing that short-term topical steroid therapy does not affect the efficacy of SLT in patients with primary open-angle glaucoma.

Trabeculectomy has been the reference standard for glaucoma filtration surgery for more than half a century. Antimetabolites used to prevent scarring can cause diffuse filtering blebs that can be uncomfortable, especially if they are high and overhanging. Mizuno and co-workers investigated the effects of overhanging blebs on corneal high-order aberrations using a wavefront analyser [12]. Overhanging blebs after trabeculectomy with a fornix-based conjunctival flap using mitomycin C resulted in an increase in high-order corneal aberrations. The proportion of cornea covered by the bleb correlated positively with the duration of the post-trabeculectomy period and with most of the high-order corneal aberrations causing visual disturbances in the late post-trabeculectomy period.

Lowering intraocular pressure remains the only clinically available treatment, and despite controlled IOP, a proportion of patients progress to blindness. Therefore, efficient neuroprotective therapies would be of great value. The mechanisms underlying retinal ganglion cell degeneration have been studied in several animal models, which, though showing different mechanisms of neuronal damage, may share some common degenerative mechanisms and genetic pathways related to ganglion cell death. Enz and co-workers used three publicly available RNA-sequencing datasets of animal models of glaucoma and screened the shared differentially expressed genes between the three glaucoma models against the Comparative Toxicogenomics Database to identify novel therapeutics [13]. Using a retinal explant model of retinal ganglion cell degeneration, the authors tested a number of these compounds to assess the therapeutic/neuroprotective effects of these drugs. This seems to be a promising approach since, with the increasing use of -omics technologies, there is a wealth of open data in the field of glaucoma that can be explored.

In summary, this Special Issue provides an overview of new treatment strategies and future goals in the management of glaucoma. It also contains new findings that may provide a starting point for new research in this important field. 
